# ANCA-Negative EGPA With Pulmonary, Cutaneous, and Neurological Manifestations in a 25-Year-Old Male: A Case Report

**DOI:** 10.7759/cureus.31753

**Published:** 2022-11-21

**Authors:** Abdullah Shehryar, Abdur Rehman, Samar Sajid, Muhammad Haseeb, Mohammad Owais

**Affiliations:** 1 Internal Medicine, Allama Iqbal Medical College, Lahore, PAK; 2 Internal Medicine, Mayo Hospital, Lahore, PAK; 3 Medicine, Dow University of Health Sciences, Karachi, PAK; 4 Internal Medicine, Jinnah Hospital Lahore, Lahore, PAK; 5 Internal Medicine, Bahria International Hospital, Lahore, PAK; 6 Medicine, Mayo Hospital, Lahore, PAK

**Keywords:** eosinophilia, vasculitis, granuloma, asthma, churg strauss syndrome, eosinophilic granulomatosis with polyangiitis (egpa), egpa

## Abstract

Eosinophilic granulomatosis with polyangiitis is a systemic vasculitis characterized by the presence of asthma, hyper-eosinophilia, and necrotizing vasculitis with extravascular eosinophilic granulomas. We report the case of a 25-year-old male who presented to the outpatient department complaining of joint aches and numbness in the hands and legs. Physical examination revealed erythematous blanchable macular rashes on palms and soles. Raynaud’s phenomenon was also observed. Lab workup revealed elevated WBC count and peripheral blood eosinophilia. Antibody tests were positive only for anti-nuclear antibodies. A diagnosis of eosinophilic granulomatosis with polyangiitis including peripheral neuropathy, arthralgia, rash, and pulmonary manifestations was established. The patient was started on a therapeutic regimen of corticosteroids and immunosuppressants, which halted the progression of the disease. Peripheral neuropathy and arthralgia also improved.

## Introduction

The first case of eosinophilic granulomatosis with polyangiitis (EGPA), also known as Churg-Strauss syndrome (CSS), was reported by Churg and Strauss in 1951 [1]. The global incidence of this disease is 2.5 cases per 100,000 adults per year [2]. EGPA is a multisystemic anti-neutrophil cytoplasmic antibodies (ANCA)-associated disorder and is characterized by eosinophilia and granulomatous inflammation, which is classically described as a triad of asthma, eosinophilia, and necrotizing vasculitis. It occurs with systemic vasculitis involving small- and medium-sized vessels. It is an extremely rare and progressive disease affecting multiple tissues and organs simultaneously [3]. Usually, upper airway involvement is frequently seen in such vasculitides [4]. The American College of Rheumatology (ACR) established six EGPA criteria and outlined the categorization criteria used to differentiate between various vasculitides. Vasculitis is labeled as an EGPA when four or more of these conditions are satisfied [1]. ACR criteria: Presence of four or more of the following: 1) peripheral eosinophilia (more than 10%), 2) asthma, 3) pulmonary infiltrates, 4) paranasal abnormalities, 5) neuropathy, and 6) extravascular eosinophilia on biopsy [5].

## Case presentation

A 25-year-old male presented to the outpatient department complaining of severe joint ache in his hands and hip joints. He had a feeling of numbness in his legs and hands for the last two months. He also had a history of chronic rhinitis and asthma for the last five years and underwent nasal polypectomy one year ago. He was on montelukast, salbutamol inhaler, and anti-histamine medication. On physical examination, erythematous blanchable blotchy macular rashes on palms and soles were observed. Raynaud’s phenomenon was also present in hands and feet.

Initial laboratory investigations showed elevated WBCs (19.1 x 10^9^/L), platelets (422 x 10^9^/L), neutrophils (88%), eosinophils (2.3 x 10^9^/L), and erythrocyte sedimentation rate (29 mm/h). There was a decline in mean corpuscular hemoglobin (31%) and lymphocytes (8%). Lipid profile showed elevated total cholesterol/high-density lipoprotein (HDL) cholesterol ratio (8.1) and a decline in HDL cholesterol (22 mg/dL). Liver function tests showed elevated aspartate transaminase (51 U/L) and serum globulin (4.4 g/dL). Renal function tests were normal. C-reactive protein (CRP) was also elevated (20.91 mg/L) (Table 1). 

**Table 1:** Initial Lab Investigations WBC: White Blood Cells; ESR: Erythrocyte Sedimentation Rate; MCH: Mean Corpuscular Hemoglobin; CRP: C-Reactive Protein; HDL: High-Density Lipoprotein; ANA: Anti-Nuclear Antibodies; ANCA: Anti-Neutrophil Cytoplasmic Antibodies; P-ANCA: Perinuclear Anti-Neutrophil Cytoplasmic Antibodies; DsDNA: Double-Stranded Deoxyribose Nucleic Acid; RF: Rheumatoid Factor.

Blood Workup			
Test	Value	Reference Range	Comment
WBC	19.1 x 10^9^/L	4-11 x 10^9^/L	Above Normal
Eosinophils	2.3 x 10^9^/L	<0.5 x 10^9^/L	Above Normal
Platelets	422 x 10^9^/L	150-400 x 10^9^/L	Above Normal
Neutrophils	88%	40-75%	Above Normal
CRP	20.91 mg/L	0.0-5.0 mg/L	Above Normal
ESR	29 mm/h	0-10 mm/h	Above Normal
MCH	31%	26-32%	Below Normal
Lymphocyte	8%	20-45%	Below Normal
Lipid Profile			
Cholesterol/HDL Cholesterol Ratio	8.1	Less Than 5.0	Above Normal
HDL Cholesterol	22 mg/dL	35-55 mg/dL	Below Normal
Liver Function Tests			
Aspartate Transaminase	51 U/L	Less Than 50 U/L	Above Normal
Serum Globulin	4.4 g/dL	1.8-3.8 g/dL	Above Normal
Antibody Tests			
ANA	Positive		
ANCA	Negative		
P-ANCA	Negative		
DsDNA	Negative		
RF	Positive		

**Figure 1:**
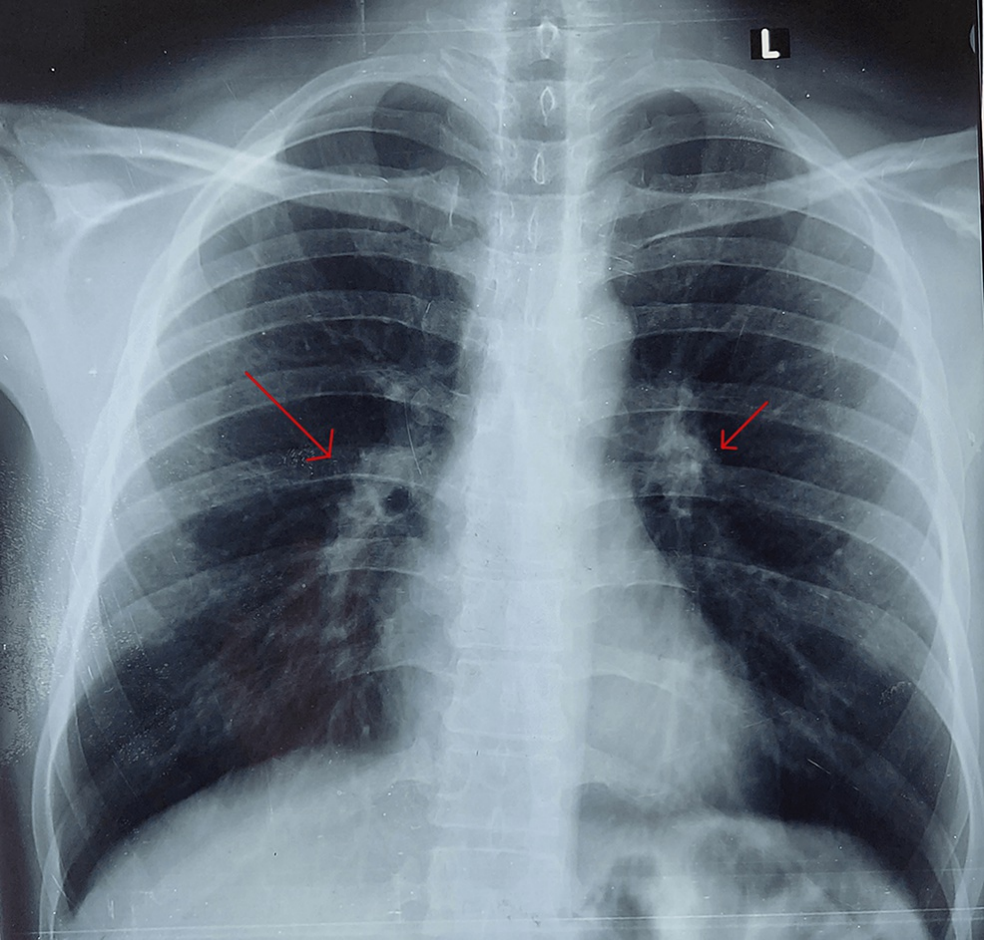
Infiltrations seen on chest X-ray, especially in the right lung and bilateral hilar involvement.

**Figure 2:**
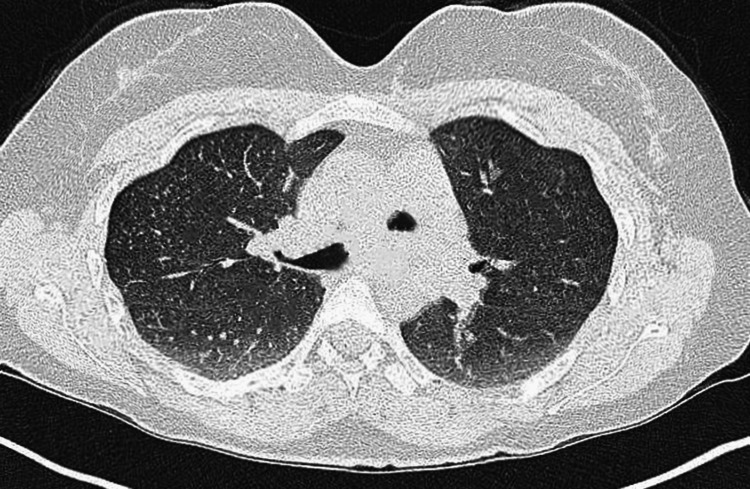
Diffuse centrilobular ground-glass nodules along the bronchovascular structures in chest CT.

**Figure 3:**
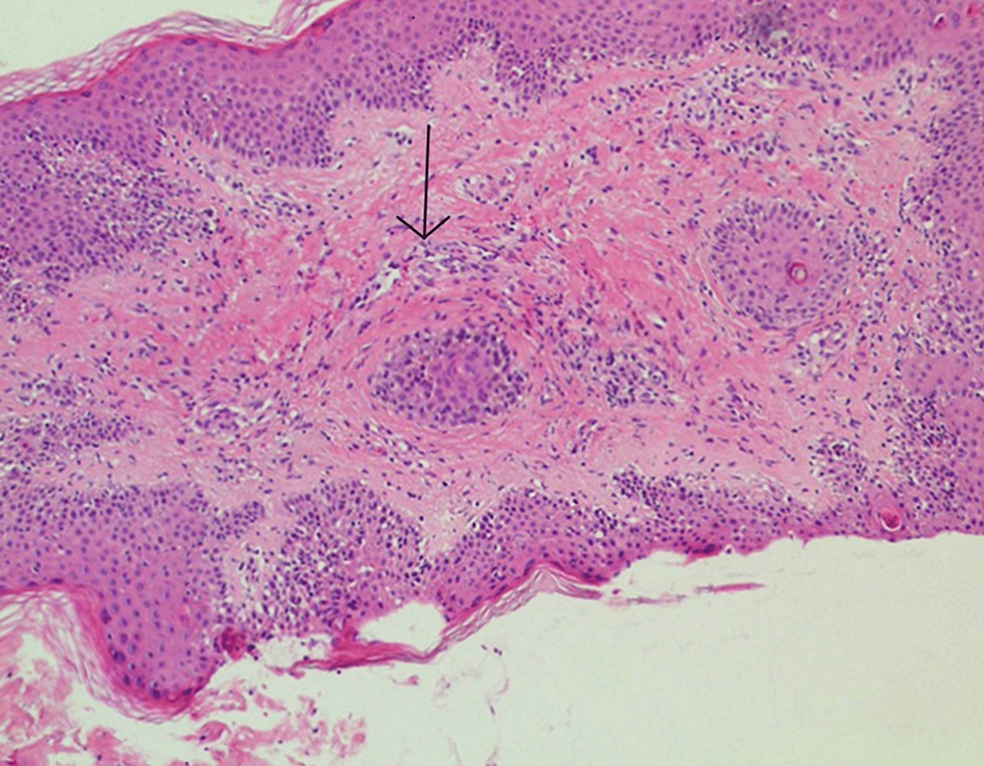
Biopsy of skin lesion showing tissue eosinophilia and granuloma (hematoxylin and eosin staining).

X-ray of the lungs showed infiltrations, especially in the right lung, and hilar involvement (Figure 1). Hence, the lungs' involvement was confirmed. The serology report indicated the presence of rheumatoid factor (RA factor). The autoimmune disorder was suspected, and thus antibody tests were conducted. Anti-nuclear antibodies (ANA) were positive with an estimated endpoint titer of 1/5120. Double-stranded deoxyribose nucleic acid (DsDNA), cytoplasmic ANCA (C-ANCA), and perinuclear ANCA (P-ANCA) were negative. The rheumatology department was consulted and a differential diagnosis of undifferentiated connective tissue disorder (UDCTD) and EGPA was established. A biopsy of skin lesions showed intense eosinophilia with granulomatous vasculitis (Figure 2). A high-resolution CT chest showed subtle centrilobular ground-glass nodules along the bronchovascular structures (Figure 3). These findings along with notable peripheral blood eosinophilia were in consistence with the diagnosis of EGPA.

The patient was started on prednisone 20 mg, methotrexate 10 mg, folic acid 5 mg, montelukast 10 mg, and hydroxychloroquine sulfate 200 mg. Peripheral neuropathy and arthralgia resolved within one month of treatment. Labs were repeated six months later showing a significant decrease in WBC count and peripheral blood eosinophilia.

## Discussion

EGPA is a type of ANCA-associated vasculitis that predominantly affects small- and medium-sized vessels of many organs simultaneously [6]. Most common manifestations in children are seen in the respiratory tract (69%), cutaneous tissue (61-62%), gastrointestinal tract (46%), cardiovascular system (46%), paranasal sinus abnormality (38%), arthritis/arthralgia (38%), and peripheral nerves (15%) [7-11]. In adults, other respiratory features also come to observation while the overall pattern of involvement is almost similar to children. The patient usually has a history of chronic asthma, longstanding rhinitis, recurrent sinusitis, otitis media, nasal obstruction, and nasal polyposis. Asthma is one of the most common associations of EGPA and has several types which include allergic asthma mediated by IgE, aspirin-exacerbated respiratory disease, exercise-induced asthma, non-allergic asthma often triggered by viral upper respiratory tract infections or no apparent cause, and cough variant asthma [12].

The exact pathogenesis of EGPA is not known but probably results from the mutual effects of B and T lymphocytes besides eosinophils [13]. This means autoantibodies and T cells act against various tissues of the body precipitating the aforementioned symptoms. ANCA is negative in most EGPA patients [7]. Patients with restricted disease, or disease that only affects the upper and lower respiratory tracts and does not affect the kidneys, are more likely to have ANCA-negative instances. ANCA-positive cases are usually accompanied by elevated eosinophils, which might mediate organ damage [14]. Our patient was ANCA negative with high levels of eosinophils in the bone marrow and peripheral blood with respiratory, cutaneous, neural, and skeletal manifestations. The possible mechanism of organ injury is eosinophil-associated vascular occlusion leading to ischemia and eosinophil-associated tissue damage [12]. In the vasculitic phase of the disease, histopathology shows eosinophilic infiltration of the vessel walls and necrotizing eosinophilic granuloma in extravascular tissue [15].

The clinical picture of EGPA is characterized by a history of rhinosinusitis, chronic asthma, and peripheral eosinophilia, and hence, its diagnosis revolves around the evaluation of these features. Clinical signs, routine laboratory tests, echocardiography, and biopsy results help construct a complete picture of the disease in such patients. With further progression of the disease, multiple organs and tissues get affected as well as small- and medium-sized vessels. So, organ-specific evaluation techniques are used to determine the extent of the disease. The best-known lab hallmark of the disease is peripheral blood eosinophilia. Other lab findings may also be present but are non-specific such as changes in erythrocyte sedimentation rate (ESR), CRP, and normocytic normochromic anemia [16]. CT sinuses and CT chest are beneficial in determining sinus and respiratory involvement, respectively. Sural nerve biopsy indicating axonal degradation and perineural eosinophilia is the gold standard for diagnosing peripheral neuropathy associated with EGPA [17]. MRI is the imaging modality of choice for observing cardiac manifestations. Eosinophilia is also observed in skin lesions on biopsy. Recently, the American College of Rheumatology (ACR) and the European Alliance of Associations for Rheumatology have published a new set of EGPA diagnostic criteria having a sensitivity of 85% and a specificity of 99% [18]. It is meant to be used when a diagnosis of small-vessel or medium-vessel vasculitis has been made. For EGPA categorization, a total score of 6 or above is required.

Earlier diagnosis and prompt evaluation of the stage of the disease are key to treatment. A therapeutic regimen involving corticosteroids and immunosuppressants is administered. Corticosteroids are beneficial for controlling eosinophilia, inflammation, and symptoms of asthma. Immunosuppressive drugs deal with the autoimmune factors, which play an etiological role in the progression of the disease. Doses vary and depend upon the stage of the disease. Prednisone, methylprednisolone, methotrexate, and azathioprine are the most commonly prescribed drugs. Recent studies have revealed that mepolizumab (IL-5 monoclonal antibody) and omalizumab are successful in controlling refractory EGPA [19,20]. These therapeutic regimens are used to control the symptoms; however, the ideal treatment for the disease is still unknown.

Pharmacological treatment is based on early recognition, irrespective of the etiology and mechanism of the disease. Patients with EGPA are able to achieve disease remission with corticosteroids and now anti-IL-5 therapy. Treatment with immunomodulators with the use of plasma exchange or intravenous immunoglobulin (IVIG) is reserved for more refractory cases. It responds well to treatment but is also characterized by a high remission rate and a lingering persistence of difficult-to-control asthma and systemic manifestations affecting the quality of life.

## Conclusions

CSS or EGPA is a pleiotropic systemic vasculitis with a dual face of manifestations based on eosinophilic damage or ANCA-associated small- and medium-vessel injury. This dichotomy has made it challenging to identify a gold standard for diagnosis and has also made prognosis somewhat variable. Patients who test negative for ANCA are younger at diagnosis, suffer from persistent asthma, and have cardiac involvement. Corticosteroids, immunosuppressants, and monoclonal antibodies are effective in preventing this disease from progression, sometimes even leading to remission.
